# South Africa’s experience with provision of quality HIV diagnostic services

**DOI:** 10.4102/ajlm.v5i2.436

**Published:** 2016-10-17

**Authors:** Natasha M. Gous, Leigh Berrie, Patience Dabula, Wendy Stevens

**Affiliations:** 1 National Health Laboratory Service, Johannesburg, South Africa; 2 National Priority Program of the NHLS, Johannesburg, South Africa; 3 Department of Molecular Medicine and Haematology, School of Pathology, University of the Witwatersrand, Johannesburg, South Africa

## Background on HIV in South Africa

South Africa is an upper–middle-income country with the second-largest economy in Africa.^1^ In mid 2015, the population was estimated at 54.96 million; 51.0% were women and 30.2% were younger than 15 years of age.^2^ The population density, age and gender structures, however, vary significantly.

South Africans have a high burden of communicable diseases such as HIV and tuberculosis, as well as non-communicable chronic diseases such as diabetes, hypertension and cancer.^3^ Life expectancy has seen progressive increases over the years, in part due to the rapid and effective scale-up of HIV and tuberculosis care in the country.^2,4^ In 2015, life expectancy was 60.6 years for men and 64.3 years for women.^2^ However, maternal and child mortality rates are relatively high compared to other middle-income countries.^4,5^

The latest UNAIDS estimates for 2015 indicated that almost 7 million [6.7–7.4 million] South Africans were living with HIV.^6^ In the 15–49-year age group, prevalence was as high as 19.2%, with women the worst affected.^6^ In the 0–14-year age group, 240 000 children are estimated to be living with HIV.^6^ Antiretroviral therapy coverage is expanding, with approximately 3.4 million South Africans currently receiving antiretroviral therapy and close to 4 million predicted to be on antiretroviral therapy in the 2016 and 2017 fiscal year.^3^

## Current laboratory infrastructure and HIV-related testing in South Africa

Healthcare in South Africa is two-tiered, consisting of a public sector serving over 80% of the population and a smaller private sector catering to the middle- and upper-income population, largely through medical insurance. The South African National Department of Health (NDoH) has overall responsibility for healthcare, but is particularly responsible for the public sector. South Africa is divided into nine provincial Departments of Health; each is responsible for managing and providing comprehensive healthcare services through a district-based model.^1^ An approximate 4420 primary public healthcare facilities are available, of which 3991 provide HIV treatment services.^1,7^

Diagnostic testing in the public sector is the mandate of the National Health Laboratory Service (NHLS), the largest pathology service provider in the country. The NHLS is a national public entity established through the amalgamation of a number of public-sector laboratory service providers and various Provincial Department of Health laboratories.^8^ The NHLS serves more than 80% of the population through a network of over 260 laboratories throughout the nine provinces.

**FIGURE 1:**
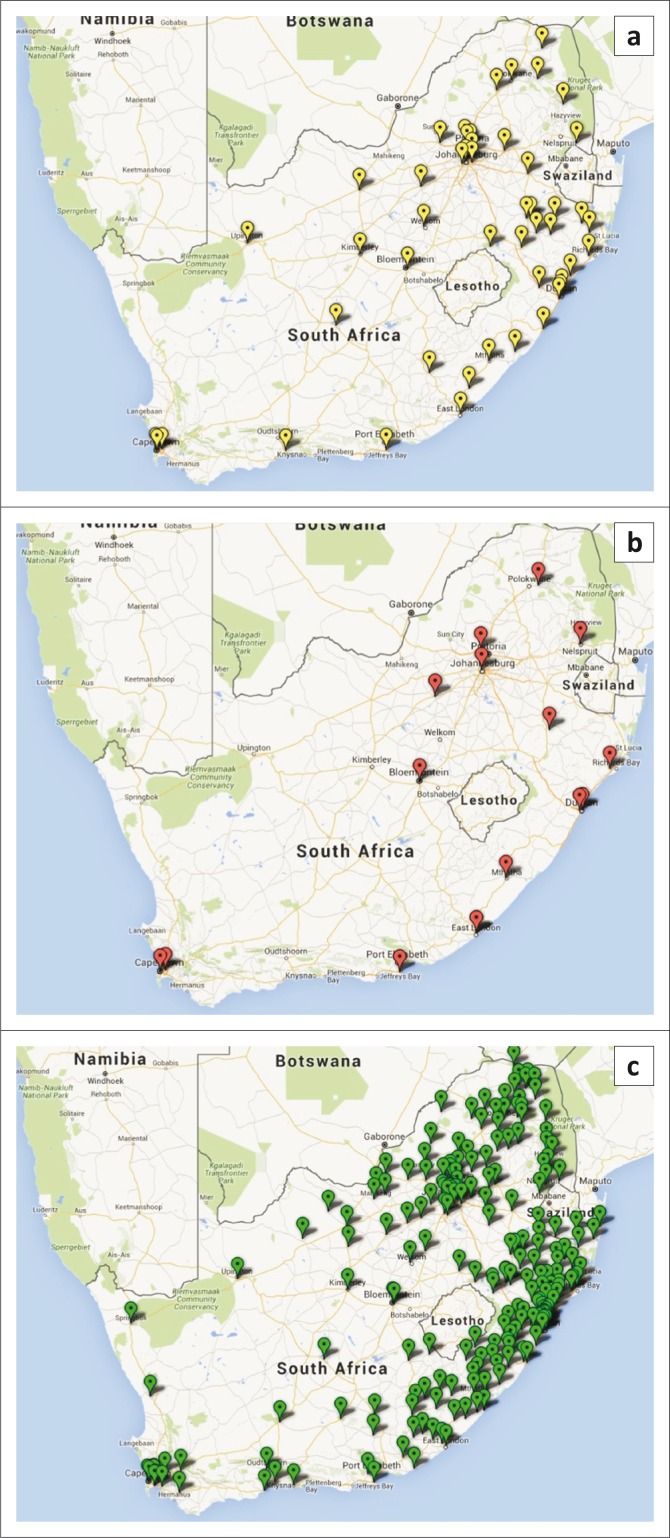
GIS map of testing laboratories implemented by the National Priority Program throughout South Africa. There are currently (a) 52 CD4 (yellow), (b) 16 viral load (red) and (c) 211 GeneXpert MTB/RIF (green) testing laboratories.

The National Priority Program was established in 2010 to provide support to the NHLS and NDoH through the management, coordination, standardisation and implementation of a number of National Programmes, including HIV viral load, early infant diagnosis (EID), tuberculosis, CD4 and, more recently, HIV drug resistance. The National Priority Program now supports 16 regional laboratories for HIV viral load testing, nine laboratories for EID, 52 regional laboratories for CD4 testing, 211 GeneXpert^®^ MTB/RIF testing sites and five HIV drug resistance testing laboratories (Figure 1).

Further infrastructure supporting the NHLS programme includes significant investment into the NHLS laboratory information system using TrakCare or DisaLab, to which all analysers within the NHLS are interfaced. All national test results are collected centrally and archived within a single central data warehouse, which is a large server able to store, manage and analyse all laboratory information system data from all tests generated.

For CD4 testing, the routine method employed is Pan*Leuco*gating, a cost-effective technology developed within South Africa and licensed to Beckman Coulter.^9,10^ Approximately 3.6 million Pan*Leuco*gating CD4 tests were performed within the NHLS in 2015.

**FIGURE 2:**
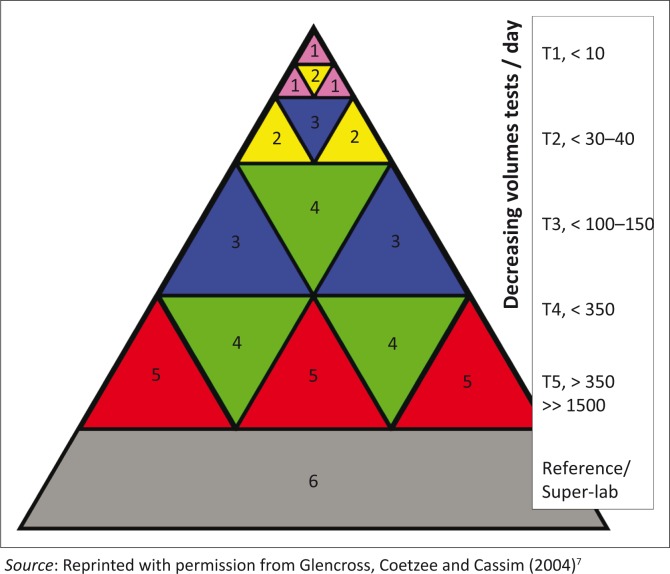
Integrated Tiered Service Delivery Model. The proposed NHLS CD4 tiered laboratory network structure comprises six service tiers, which support decreasing service test volumes in increasingly remote sites. Tier 1 (servicing a single site) and Tier 2/‘POC Hub’ (servicing up to 10 remote clinics) utilise multiple POC technologies to extend laboratory services (HIV, tuberculosis) in remote, hard-to-reach areas, beyond a reasonable distance to a Tier-3 laboratory; Tier 3 represents a community laboratory that serves > 10 < 50 clinic sites; Tier 4 and Tier 5 are regional laboratories or ‘metro’ centralised laboratories performing high-volume testing; Tier 6 represents coordinated, harmonising national support from an expert team or reference laboratory.^7^

South Africa has also evaluated the Alere Pima™ CD4 technology and other point-of-care (POC) devices.^11,12,13^ A number of Pimas have been implemented by non-government organisations^14^ and Department of Health facilities in certain provinces for pilot studies, but the South African NHLS has not yet adopted Pima or any other POC technology for wide-spread implementation. This has mainly been due to current reliable CD4 services with good laboratory turnaround times^7^ and the prohibitive cost of implementing widespread CD4 testing at the POC.^15^ However, with increasing testing demands, expansion of CD4 testing services is required. A newly-proposed CD4 testing model, the Integrated Tiered Service Delivery Model^7^ (Figure 2), provides a service delivery system that enables high-volume testing on one end, whilst integrating and extending laboratory services into small laboratories with existing infrastructure, to rapidly scale up services, to lower volume sites on the other end. Placement of testing equipment is therefore, in accordance with service needs; in a community laboratory, for example, small automated equipment is placed to test 100–150 samples per day, covering the service needs of all the local clinics in the area,^7^ whereas in very remote sites, without any reasonable access to a laboratory and where less than 30 tests per day are needed, multiple POC technologies can be used to supplement and extend laboratory services, facilitating total service coverage^7^ (Figure 2).

The Integrated Tiered Service Delivery Model is currently being implemented in the NHLS^7^ and has demonstrated success^16^ in substantially reducing turnaround times of reporting in remote areas of South Africa, thus ensuring total service coverage and rapid turnaround times, irrespective of where services are provided^7^ (Figure 2). Costs of the Integrated Tiered Service Delivery Model are contained and remain fixed across a network, whilst still providing reasonable access to service. Although the decentralised approach using POC (at the Tier 1 or Tier 2 level) has been shown to cost five to seven times more than a conventional laboratory-based service,^15^ these higher costs can be cross-subsidised by the majority of national service requirements (in South Africa > 90%) being met by significantly more cost-efficient conventional laboratories^7,15^ at Tiers 3, 4 and 5.^7^ The Integrated Tiered Service Delivery Model approach further enables a network for technical and quality support for lower tier sites, with lower tier sites supported within a defined locality by the nearest higher tier sites.

HIV viral load testing within the NHLS relies on centralised, high throughput, laboratory-based testing. The tender is awarded every three years through a highly competitive selection process and for the last two rounds has used two suppliers, namely Abbott Molecular and Roche Molecular Diagnostics. Within the 16 HIV viral load laboratories nationwide, 13 laboratories utilise either the Roche COBAS^®^ Ampliprep/COBAS^®^ TaqMan^®^ version 2 or the high throughput cobas^®^ 8800/6800 systems and three use the Abbott *m*2000 Realti*m*e™ HIV-1 system. All instruments are fully automated, interfaced, real-time platforms which facilitate a faster result turnaround time. In 2015 alone, over 3.5 million viral load tests were performed and this number is anticipated to increase to almost 5 million if testing targets are met for the next fiscal year (April 2016–March 2017).

In addition to scaling up centralised testing, the NHLS is also considering adopting a tiered viral load testing model using a combined approach of high throughput, mid-throughput and POC platforms and is embarking on a pilot project to evaluate the feasibility of such a model in collaboration with the NDoH.

EID is performed in nine laboratories on the Roche COBAS Ampliprep/COBAS TaqMan platform using the qualitative COBAS Ampliprep/COBAS TaqMan HIV-1 Test, v2. During 2015, approximately 450 000 HIV PCR tests for EID were performed. Due to changes in the clinical algorithm, which includes birth testing and follow-up testing to avert early mortality prior to three months of age, these numbers should theoretically increase significantly and POC technologies may have a place for niche testing, but will require further investigation.

The South African NDoH estimates that 60% – 70% of all HIV-positive persons are also co-infected with tuberculosis.^3^ Thus, together with the NHLS, the Department of Health have been global leaders in rolling out GeneXpert^®^ MTB/RIF testing in South Africa. A total of 314 GeneXpert instruments of varying sizes have been placed in 211 sites, in both urban and rural settings, with expansion of the programme to special risk populations such as correctional facilities and peri-mining communities. In future, GeneXpert^®^ MTB/RIF testing laboratories may be used for decentralisation and expansion of viral load testing services.

## Current quality assurance framework and policy for HIV testing in South Africa

The NHLS has implemented a Quality Management System in compliance with various standards (ISO 15189, ISO 17025, ISO 9001 and ISO 17043) and the competence of all medical testing laboratories is, therefore, in accordance with the relevant ISO standard and guidelines for Good Laboratory Practice. Clinical Pathology laboratories operate according to the requirements of ISO15189:2012.

The NHLS National Quality Assurance Division is the national quality-related policy-setting body and is responsible for establishing, documenting, implementing, maintaining and continually improving the quality management system. The National Quality Assurance Division manages the in-house production and distribution of External Quality Assessment (EQA) material. These proficiency testing schemes are offered both internally and externally, to private laboratories in the country and also to 23 countries outside of South Africa, in several pathology disciplines, and are operated in accordance with ISO/IEC 17043:2010. The National Quality Assurance Division is also responsible for ensuring that NHLS laboratories are accredited by the South African National Accreditation System through implementation of standard requirements, including participation in EQA programmes/proficiency testing and auditing against ISO 15189 for medical testing laboratories.

CD4 laboratories participate in the NHLS EQA programme and Beckman Coulter 3-IQAP and also monitor internal quality measures (flow count rates) to ensure ongoing excellence of service.

All viral load testing laboratories participate in the Quality Control for Molecular Diagnostics EQA programme and Centers for Disease Control Dry Test Tube programme; both programmes are coordinated by the NHLS Quality Assurance Division. EID testing laboratories participate in the NHLS Dried Blood Spot EQA Program.

Specifically for the CD4, HIV and tuberculosis programmes, the National Priority Program provides monitoring through:

- Monthly meetings with suppliers to identify problem areas, monitor monthly turnaround times, stock control, instrument breakdowns and throughput.
- Site monitoring, including monthly indicator reports detailing test volumes, errors and training needs and which are reviewed and actioned accordingly.
- For HIV viral load, a process of continuous quality monitoring is used through remote connectivity:
  ◦ The use of the Abbott *m*View software for all HIV viral load laboratories utilising the Abbott platform.
  ◦ The use of remote connectivity software (Axeda) for all HIV viral load laboratories utilising the Roche platform.
  ◦ The use of an antiretroviral therapy dashboard for continuous monitoring of laboratory performance. This dashboard was developed together with the NHLS Central Data Warehouse and generates monthly reports for both internal and external stakeholders in terms of test volumes and result ranges from national and provincial down, to district level for HIV viral load, CD4 and EID.
- Laboratory site visits and assistance for accreditation.

## National quality assurance programme for point-of-care testing in South Africa

A final policy draft is being vetted for quality assurance of POC testing and the NHLS will play a pivotal role in the management and support of POC testing services to ensure it performs to the same quality standards as current diagnostic testing. The NHLS should take full responsibility for implementation of technology, training, monitoring and evaluation, procurement and stock control.^7^ POC testing sites will implicitly follow ISO 22780 and NHLS will manage relevant accreditation procedures for sites performing POC testing.^17^

A key component of the quality assurance programme will be the inclusion of internal and external quality control procedures and management. The provision of quality assurance schemes for POC testing will be the responsibility of the NHLS, which already has the capacity, expertise and proven experience to establish and implement new programmes.

As an example, the NHLS assisted the NDoH in ensuring the quality of the approximate 8 million rapid HIV tests performed per year, through development of materials, test kit monitoring and training. This included development of a quality assurance plan and quality improvement programme in collaboration with the Centers for Disease Control, which has been adopted by the NDoH. As part of these activities, post-market surveillance of rapid-test kit lots is conducted prior to national release. The quality assurance programme for rapid HIV testing is still an area in need of improvement. Some of the key challenges experienced during implementation of the rapid HIV testing quality assurance programme included: staff not implementing what was taught during training; lack of understanding of quality management principles; staff not adhering to quality assurance testing procedures due to high workload; and lack of knowledge transfer following training.

Many of these challenges have been overcome using the following strategies:

- Initiation of ‘Train-the-trainer’ workshops.
- Development of a draft quality plan which is made available to sites.
- Training of Provincial coordinators who have overall site management and can follow up on problem sites.
- Introducing more follow-up site visits to check compliance.

The GeneXpert^®^ MTB/RIF Dried Culture Spot EQA programme, developed in collaboration with University of the Witwatersrand in 2011,^18,19^ is another example of NHLS expertise. The Dried Culture Spot EQA has become an integral component to the GeneXpert^®^ MTB/RIF programme and now supports ~391 sites in 24 countries. The material is manufactured and distributed in-house and is easy, safe, stable and cost effective. The programme is supported by an automated, real-time reporting and quality monitoring web-based tool, TBGx Monitor™ (www.tbgxmonitor.com), which remotely collects and analyses EQA data by uploading the data from individual GeneXpert modules. This programme has also shown success in non-laboratory users^18^ and is being expanded to other molecular tuberculosis diagnostic platforms^20^ as well as adaptation to other diseases. A similar quality assurance model will likely be developed for POC technologies.

### Conclusion

If POC testing is expected to improve and support diagnostic and clinical services in South Africa, the laboratory needs to play a major role in ensuring success of the programme through a POC quality assurance framework, much the same as for laboratory testing. The NHLS has demonstrated proven success in conceptualisation and implementation of quality management systems for national programmes and will adopt a similar strategy for POC testing through the Integrated Tiered Service Delivery Model.
